# Intestinal obstruction by a phytobezoar in a patient with a history of gastroplasty

**DOI:** 10.1590/0100-3984.2017.0157

**Published:** 2019

**Authors:** Luiz de Abreu Junior, Gustavo Garcia Marques, Ingredy Tavares da Silva, Flávia Munhos Granja, Marcelo Zindel Salem

**Affiliations:** 1 Grupo Fleury, São Paulo, SP, Brazil.; 2 Universidade São Camilo, São Paulo, SP, Brazil.; 3 Hospital São Luiz - Rede D'Or, São Paulo, SP, Brazil.

*Dear Editor,*

**Figure 1:**
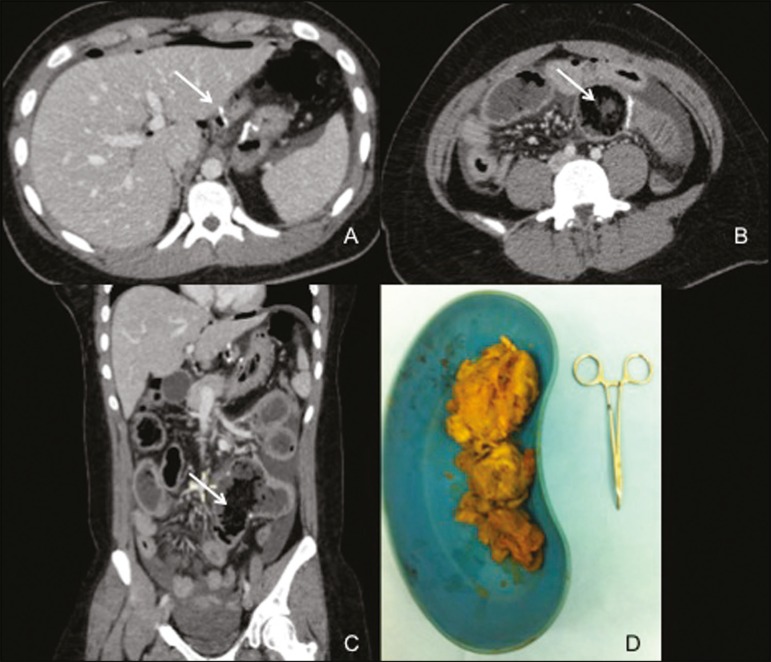
CT of the abdomen and pelvis, with intravenous contrast. **A:** Axial sequence showing signs of gastroplasty (arrow) and a small amount of perisplenic fluid. **B:** Axial sequence showing distension of the jejunal loop related to enteric anastomosis, highlighting the accumulation of material with low-grade intraluminal attenuation, corresponding to a phytobezoar (arrow). **C:** Coronal reconstruction confirming the signs of intestinal obstruction and again showing significant distention of the small intestine loop that participates in the enteric anastomosis, containing an accumulation of material with low attenuation (phytobezoar, arrow). **D:** Surgical specimen. Material removed from within the jejunal loop related to the surgical anastomosis, characterized by a bolus of undigested agglomerated vegetable fiber (a jackfruit phytobezoar).

We report the case of a 28-year-old female patient with a one-year history of gastroplasty who was suffering from abdominal pain one day after eating a large amount of jackfruit. The physical exam revealed diffuse pain on palpation, positive abrupt decompression and absence of bowel sounds. Computed tomography (CT) of the abdomen and pelvis showed signs of intestinal obstruction. Intraoperatively, intraluminal content, consistent with a phytobezoar (a jackfruit "bolus"), was observed impacting the distal anastomosis of the gastric bypass (Figure 1).

A bezoar is a mass of exogenous undigested material that accumulates in the gastrointestinal tract, usually in the stomach or ileus, and causes intestinal obstruction^(^^1^^)^. Bezoars are associated with predisposing factors such as poor mastication, psychiatric disorders, and impaired gastric motility. 

Bezoars are classified, according to their composition, as phyto bezoars (composed of vegetable fibers), lactobezoars (composed of milk), or trichobezoars (composed of hair). Phytobezoars account for 40% of all bezoars and are composed of materials of vegetable origin that human beings cannot digest (seeds, peels, roots, etc.); they develop through a multifactorial process. Individuals with a greater propensity to develop phytobezoars include not only vegetarians but also individuals who do not chew their food well, those with impaired gastric motility, and those with hypochlorhydria, as well as those who have undergone gastrectomy. A history of gastric surgery is a risk factor because it reduces the surface area of the stomach and acid secretions, causing inadequate digestion and allowing larger masses of agglomerated material to pass into the small intestine^(^^1^^-^^3^^)^. Phytobezoars can also occur in patients who have had bariatric surgery. In addition to the aforementioned factors, nonabsorbable sutures can act as vegetable fiber hooks, resulting in a bolus that forms in the anastomosis. Phytobezoar formation evolves to intestinal obstruction in 60% of cases. 

CT has become the imaging examination of choice for the diagnosis of acute abdominal obstruction, because it is a rapid method that produces high-resolution images to confirm the obstructive scenario, often making it possible identify the etiological factor. On CT, intestinal obstruction is characterized by dilated proximal intestinal loops (with a caliber > 2.5 cm), distal loops that are collapsed or are proportionally smaller than the proximal loops, and intraluminal air-fluid levels^(^^2^^,^^4^^)^.

The diagnosis of a phytobezoar should be considered in cases of intestinal obstruction when there is an obstructive intraluminal focal mass that is of low density and contains images suggestive of air bubbles^(^^2^^,^^4^^)^. Modifying the amplitude and centering of the imaging window can facilitate the identification of aspects characteristic of a bezoar, which has been described as looking like "a lump of bread". The differential diagnoses of a phytobezoar include foreign bodies, abscesses, and a worm bezoar. When the mass is seen in a colonic loop, a diagnosis of fecaloma should also be considered.

Jackfruit is common in the northern and northeastern regions of Brazil. Its potential to form phytobezoars is due to its large concentration of fibers and other components such as calcium, phosphorous, and iron^(^^5^^)^.

When assessing intestinal obstructions in patients who have undergone bariatric surgery, radiologists should be aware of the possibility of a bezoar as the cause. Preoperative clinical suspicion optimizes the surgical results.
